# DNA barcoding identifies cryptic animal tool materials

**DOI:** 10.1073/pnas.2020699118

**Published:** 2021-07-12

**Authors:** Matthew P. Steele, Linda E. Neaves, Barbara C. Klump, James J. H. St Clair, Joana R. S. M. Fernandes, Vanessa Hequet, Phil Shaw, Peter M. Hollingsworth, Christian Rutz

**Affiliations:** ^a^Centre for Biological Diversity, School of Biology, University of St Andrews, St Andrews KY16 9TH, United Kingdom;; ^b^Royal Botanic Garden Edinburgh, Edinburgh EH3 5LR, United Kingdom;; ^c^The Fenner School of Environment and Society, The Australian National University, Canberra, ACT 2600, Australia;; ^d^Institut de Recherche pour le Développement, Centre de Nouméa, 98848 Nouméa, New Caledonia, France

**Keywords:** animal construction behavior, DNA barcoding, nest building, New Caledonian crow, tool use

## Abstract

Some animals fashion tools or constructions out of plant materials to aid foraging, reproduction, self-maintenance, or protection. Their choice of raw materials can affect the structure and properties of the resulting artifacts, with considerable fitness consequences. Documenting animals’ material preferences is challenging, however, as manufacture behavior is often difficult to observe directly, and materials may be processed so heavily that they lack identifying features. Here, we use DNA barcoding to identify, from just a few recovered tool specimens, the plant species New Caledonian crows (*Corvus moneduloides*) use for crafting elaborate hooked stick tools in one of our long-term study populations. The method succeeded where extensive fieldwork using an array of conventional approaches—including targeted observations, camera traps, radio-tracking, bird-mounted video cameras, and behavioral experiments with wild and temporarily captive subjects—had failed. We believe that DNA barcoding will prove useful for investigating many other tool and construction behaviors, helping to unlock significant research potential across a wide range of study systems.

There is increasing interest in the plant materials selected by nonhuman animals to manufacture foraging tools and constructions (1, 2). Animals’ raw-material preferences can affect the structural and functional properties of artifacts, and, in some cases, appear to be socially transmitted, contributing to rudimentary material “cultures” (3–5). Two complementary approaches are available for identifying plant materials used by wild animals: direct observation of manufacture behavior (“animal-centered”) and examination of artifacts in isolation from the behavior that created them (“artifact-centered”). The latter, adopted by necessity in archaeology, is particularly useful when animals cannot be habituated or are otherwise difficult to observe, but can present considerable challenges. Artifacts are often heavily processed (lacking features that aid identification, such as leaves or flowers), may be physically distanced from the raw materials from which they were produced (because the animal transported them), and may comprise a complex assemblage of materials from different sources (such as in bird nests). In these cases, material identification has so far relied on expert knowledge, which may be difficult and expensive to acquire (6). Here, we demonstrate that DNA barcoding—the use of standardized DNA regions to identify organic material to species level (7)—provides a robust, cost- and time-efficient solution to these problems.

New Caledonian crows (*Corvus moneduloides*) are renowned for their ability to manufacture complex foraging tools (8). When making a hooked stick tool, they select a forked plant stem, remove a suitable branch, trim off any leaves and twiglets, and often refine the tool by sculpting the remains of the nodal joint into a neat terminal hook, stripping bark near the functional end, and bending the tool shaft (9). These processing steps substantially alter the appearance of the plant material (Fig. 1*A*). Importantly, properties of the raw material affect the morphology of the resulting tools, which in turn affects foraging efficiency (9–11). New Caledonian crows are highly selective when choosing plants for hooked stick tool manufacture: we recently discovered that three study populations target different species despite living just a few kilometers apart (12). While we managed to identify raw materials at two sites (site-1 and site-2), we failed at the third (site-3), even after employing a wide range of well-established field methods aimed at observing tool manufacture directly (Fig. 1 *B* and *C* and *SI Appendix*).

**Fig. 1.**
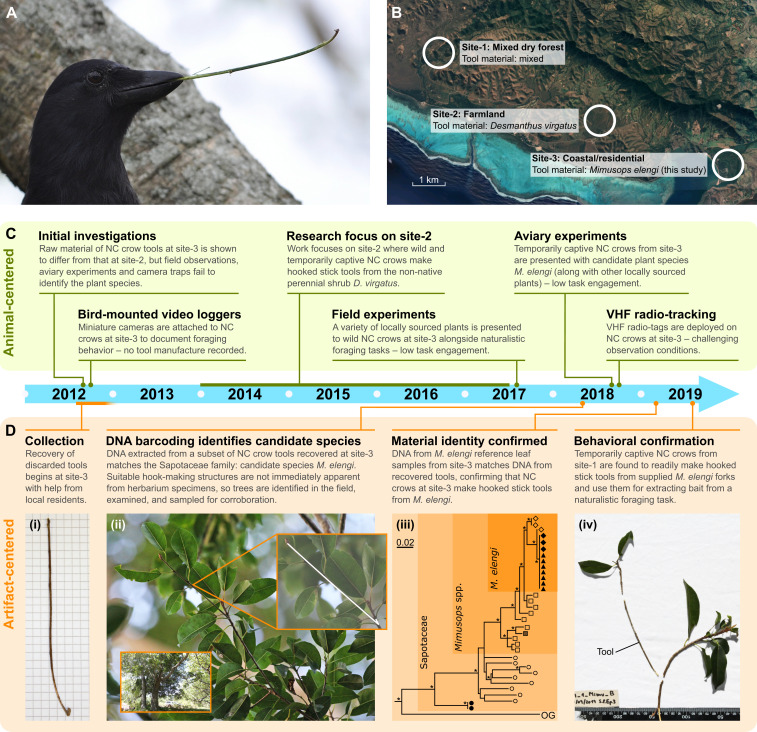
Identifying the raw material used by wild New Caledonian (NC) crows for manufacturing hooked stick tools. (*A*) NC crow holding a hooked stick tool manufactured from *Desmanthus virgatus* at site-2. (*B*) Satellite photograph showing study sites on the west coast of Grande Terre, New Caledonia. Map image credit: © 2019 Google Maps/CNES/Airbus, TerraMetrics, Data SIO, NOAA, U.S. Navy, NGA, GEBCO. (*C*) Timeline of animal-centered approaches employed while attempting to identify the tool material used at site-3, with short explanations for their limited success. (*D*) Timeline of the artifact-centered, DNA barcoding approach which ultimately led to successful material identification. (*i*) Hooked stick tool recovered at site-3 (5 × 5 mm background). (*ii*) *M. elengi* trees, with close-up of a forked terminal branch, which would be suitable for tool manufacture (shape [in white] of potential tool overlaid on image). (*iii*) Simplified maximum-likelihood ITS phylogenetic tree detailing clustering of tool samples and a subset of reference data (data for the full tree are deposited in Dryad). Symbols denote samples from *M. elengi* (diamonds), crow tools (triangles), *Mimusops* spp. (squares; from top: *M. zeyheri*, *M. caffra*, *M. comorensis*, *M. obovata*, *M. kummel*, *M.* sp., *M. coriacea*, *M. lecomtei*, *M. perrieri*, *M. membranacea*), and Sapotaceae spp. (circles; from top: *Tieghemella heckelii*, *Autranella congolensis*, *Labourdonnaisia* spp., *Faucherea* spp., *Labramia* spp., *Manilkara* spp., *Baillonella toxisperma*, *Vitellaria paradoxa*, *Vitellariopsis* spp. and two samples from *P. cinerea* collected in this study). OG is an outgroup (*Sarcosperma laurinum*). Filled symbols denote samples collected from the study site, open symbols denote those from outside of New Caledonia accessed through GenBank, and hatching denotes a species potentially introduced to New Caledonia (but not sampled there). Asterisks indicate >70 bootstrap support, and the scale bar shows substitutions per site. A single *Manilkara hexandra* sequence from GenBank (JX856473), which resolved with *Mimusops*, is omitted here, as it was most likely a misidentification (all other sequences from this genus clustered elsewhere, as shown). (*iv*) “Refit” of a hooked stick tool made from *M. elengi* material by a temporarily captive crow from site-1: the tool is displayed along with stem and plant debris which were discarded by the crow during the manufacture process (scale in millimeters).

Given the importance of identifying the crows’ preferred plant species at site-3 for our overall research program, we tried an innovative artifact-centered approach (Fig. 1*D*). We extracted DNA from seven hooked stick tools recovered at the site during 2016–2017 and amplified two DNA barcoding regions: *trnL-UAA* [∼500 bp (13)] and internal transcribed spacer (ITS) [∼600 bp (14)]. All samples produced identical haplotypes. Comparison against the National Center for Biotechnology Information (NCBI) Nucleotide nonredundant database indicated the tool samples belonged to the family Sapotaceae, most likely the genus *Mimusops* or *Manilkara* (*trnL*: >98% identity; ITS: >95% identity). The ITS region exhibited greater resolution and indicated *Mimusops elengi* as a candidate (96 to 99% identity). With a putative source identified, we collected reference leaf samples of *M. elengi* and *Planchonella cinerea*, the only closely related species known to occur locally, and analyzed them using the same method. The tool samples and *M. elengi* reference samples produced identical haplotypes for both DNA barcodes. Furthermore, the maximum likelihood phylogenetic tree for ITS produced well-supported lineages that clustered tool samples and *M. elengi* reference samples together, within a wider clade of non-New Caledonian *M. elengi* sequences (Fig. 1 *D*, *iii*). *P. cinerea* reference samples clustered within the Sapotaceae, but outside the *Mimusops* genus, as expected, confirming that the crow tools from site-3 were made from *M. elengi*. We subsequently verified that wild-caught, temporarily captive New Caledonian crows readily manufacture hooked stick tools from this material (this work was conducted at site-1, since birds from site-3 proved too difficult to work with in field aviaries).

The use of DNA barcoding has led to an important breakthrough for our research program. Reliable raw-material identification is key to uncovering the drivers of the striking regional divergence we observed in an important aspect of New Caledonian crows’ hooked stick tool-making behavior (15). Specifically, with a set of three study populations established, and the ability to conduct rapid surveys across additional replicate sites, it will now be possible to examine whether crows’ raw-material preferences are related to environmental variation in the availability of different plant species and foraging opportunities; for example, birds may simply use a locally common tool material, or they may choose a material that is mechanically well suited to targeting local prey resources (in fact, DNA barcoding could potentially also be used to determine prey identity, using trace DNA left on tool tips). Such ecological work is of critical importance for informing our understanding of technological (cultural) evolution in this model species (16).

Perhaps more importantly, we believe that genetic approaches will be useful for many other study systems where traditional observational methods are not feasible or would cause undue disturbance, and/or where plant materials are routinely transported or heavily modified. For example, DNA barcoding could facilitate the identification of raw materials used by chimpanzees (*Pan troglodytes*) for making tools for termite fishing and other tasks, avoiding the need to regrow plants and curate herbarium specimens (4–6). We also envisage studies that identify—from small samples—individual components of complex composite structures such as bird nests and bowers, replacing time-consuming destructive investigation. There are also exciting opportunities for further methodological refinement. For example, it should be possible to recover DNA from artifacts held in museum and research collections, potentially enabling productive retrospective analyses (17). Furthermore, targeting more variable regions of the genome, such as single-nucleotide polymorphisms, could help identify, more precisely, where an animal collected plant materials (18), providing valuable information on search and transport costs. Reliable, cost- and time-efficient raw-material identification will facilitate detailed investigation of how animals source plant materials from the environment, and how the properties of these materials affect the function of the resulting artifacts.

## Materials and Methods

Methods are summarized in the main text and Fig. 1 *C* and *D*. *SI Appendix* contains extended methods, detailing our unsuccessful animal-centered (observation-focused) approaches and successful artifact-centered (DNA barcoding) approach. The latter includes DNA sequencing of samples, the search strategy and recovery of sequences from the NCBI Nucleotide nonredundant database, and the subsequent phylogenetic analyses. All sequences produced in this study are deposited in GenBank, and all data for the full maximum-likelihood phylogenetic trees (ITS and *trnL*) that were used for raw-material identification are deposited in Dryad (for details, see *Data Availability*).

## Supplementary Material

Supplementary File

## Data Availability

DNA sequence data have been deposited in GenBank (MT366813–MT366824 and MT366951–MT366962). Sequence alignments and resulting phylogenetic trees are deposited in Dryad (https://doi.org/10.5061/dryad.d7wm37q1v) (19), including GenBank accession numbers.
