# Supplementary material for: DNA barcoding identifies cryptic animal tool materials
Source: Proc Natl Acad Sci U S A. 2021 Jul 12;118(29):e2020699118. doi: 10.1073/pnas.2020699118 (PMC8307691; doi:10.1073/pnas.2020699118)
Supplement: Supplementary File [file pnas.2020699118.sapp.pdf]

PNAS
www.pnas.org

Supplementary Information for

DNA barcoding identifies cryptic animal tool materials

Matthew P. Steele, Linda E. Neaves, Barbara C. Klump, James J. H. St Clair, Joana R. S. M. Fernandes, Vanessa Hequet, Phil Shaw, Peter M. Hollingsworth, and Christian Rutz

Christian Rutz
Email: christian.rutz@st-andrews.ac.uk

This PDF file includes:

Extended methods
SI References

Extended methods

Animal-centered approaches (see Fig. 1*C* in the main text)

Initial investigations. In September and October 2012, five New Caledonian (NC) crows were caught at site-3 (for study site locations, see Fig. 1*B*), housed in temporary on-site field aviaries, and provided with assorted, locally sourced plant materials and extraction tasks (logs with drilled holes baited with small pieces of meat). None of these subjects manufactured hooked stick tools. For full details and methodology, see (1), and for husbandry protocols, see (2).

Between September and November 2012, a baited setup was presented to wild NC crows at site-3. The setup consisted of either an extraction log (presented on the ground or raised on wooden tripods), or a raised platform with a water bath and/or food bait (meat, fruit, or cat food). The setup was monitored using an autonomous camera trap (Bushnell Trophy Cam, Trophy HD, model no: 119447) that recorded 60-second-long videos at each trigger. NC crow visits to the setup were captured in 345 videos, and tool use was seen in 153 of these. Four visits contained tool manufacture episodes, in which crows sourced plant material from small shrubs immediately beside the extraction log (the plant species could not be identified). We were able to confirm that three of the tools manufactured were non-hooked sticks, but for the fourth, it was impossible to determine tool type. In all four manufactures, however, crows performed only minimal processing (e.g., removing leaves and twiglets) after detaching material from the plant and before using it as a tool. We did not observe any of the processing that is commonly associated with the manufacture of hooked stick tools, such as sculpting of the tool's functional end, bark stripping, or tool-shaft bending (3). A similar setup was used at site-3 between September and November 2013, but only a baited, raised platform was presented (no extraction logs). A total of 3,343 videos were recorded by autonomous camera traps but, since tool use was only observed in conjunction with an extraction log in 2012, we have not formally reviewed these videos.

In addition to these recordings, during occasional targeted observation sessions in a private garden at site-3 (see below), a wild NC crow was once seen manufacturing a stick tool (likely non-hooked) in a large tree, which was later identified as a Banyan (genus *Ficus*) growing around an established *Cerbera manghas* tree.

Bird-mounted video loggers. In October 2012, we fitted three NC crows at site-3 with miniature video loggers (two of these birds had been held in temporary captivity for the aviary experiments described above). Following standard protocols, the birds were sexed morphologically [all males; (4)] and their age was estimated by gape coloration [one immature and two adults; (5)]. For more details on video logger specifications and deployment methods, see (6, 7). We managed to recover two loggers: one unit was removed from the bird upon re-capture, but was badly damaged and no video footage was salvaged; the other unit, which detached as planned, had recorded video footage, and although one instance of tool use was recorded (probing with a tool in a hole in a tree), no clear view of the tool itself was captured, and it could not be established if the tool was hooked or non-hooked. No tool-manufacture episodes were recorded.

Field experiments. A baited extraction log was presented to wild NC crows at site-3 in August and September 2017 and monitored by either human observers in nearby hides or autonomous camera traps (as above). Locally sourced plant material was occasionally presented alongside the log. During a total of 12.1 hours (over nine non-consecutive days) of in-person monitoring, we observed four NC crow visits to the log and one instance of tool use. Of the 97 camera-trap videos which recorded NC crow visits, 54 contained tool use. Where crows were recorded sourcing a tool, they appeared to take sticks from the ground nearby and no tool manufacture was observed. Visits to the log were rare, presumably due to the good availability of food in surrounding gardens (e.g., chicken feed, cat food, bones left out for dogs, and coconuts cracked open by residents to feed wild birds) and the presence of stray cats.

Aviary experiments. Between September and October 2018, four NC crows (all males; one juvenile, one immature, and two adults) were trapped at site-3 and kept in temporary captivity in field aviaries (as above). To facilitate birds' habituation to aviary confinement and experimental proce-

dures, and to avoid corrupting potential local NC crow "cultures" [captive and wild birds may socially learn tool behaviors from each other that they do not naturally express; (1, 8)], we always erect field aviaries near the site of capture. After careful consideration, we made an exception on this occasion. Due to lack of suitable space and the large number of free-roaming cats and dogs at site-3, we transported crows to field aviaries at nearby site-2. In order to prevent any potential visual interaction between captive site-3 subjects and local wild crows from site-2, all aviaries were covered in a double layer of UV screen for the duration of the experiments (allowing sufficient light in but preventing a clear view). All subjects were released at site-3 where they had been caught.

Subjects were presented with a choice of plant materials for tool manufacture and a food extraction task both in their housing aviaries and in the experimental chamber. Informed by our DNA barcoding analyses of tools recovered at site-3 (see below), plants presented always included *Mimusops elengi* and, when sufficient source material was available (6 of 15 presentations), *Planchonella cinerea*, the only closely related species known to occur locally. Other plant species presented could not be identified reliably but were sourced locally and had a branching structure that was judged suitable in principle for hooked stick tool manufacture. Plants were displayed in pseudo-randomized positions standing upright on presentation logs either at ground level or raised to ~1 m on wooden tripods, to better approximate how NC crows may encounter material in larger tree species (like *M. elengi* and *P. cinerea*) in the wild. All subjects were reluctant to engage with extractive foraging tasks, and when they did, were only observed to forage with supplied sticks and non-hooked stick tools they had manufactured from *Desmanthus virgatus*. One individual briefly used a small twig of *M. elengi* to probe in its metal ID ring.

NC crows normally habituate very quickly to our field aviaries, and readily participate in behavioral experiments. On this occasion, our impression during husbandry activities and experimental work was that birds were unsettled. We suspect that transportation between sites, and unfamiliarity with site-2 (including the calls of unfamiliar wild crows visiting the aviaries), may have contributed to the subjects' lack of engagement with tasks. These observations caution that NC crows should not be routinely translocated for behavioral experiments.

VHF radio-tracking. Six NC crows (two females, four males; one juvenile, two immatures, and three adults) were caught at site-3 between September and October 2018 and fitted with VHF radio-tags (picopips, Biotrack Ltd., UK; tag mass: 1.8–2.1 g). Tags were attached with epoxy adhesive to the base of the birds' two central tail feathers, and the antenna was held in place along the shaft of one of these with two to three knots of dental floss fixed with superglue. Four of the six individuals released with tags had previously been held in field aviaries and tested in behavioral experiments (see above).

Following release, crows were allowed to habituate to the tag for at least one full day before tracking commenced. The order in which we searched for tagged crows each day was pseudo-randomized. At the start of each tracking session, a sweep over the entire study site was carried out by car and, if an individual's signal was detected, we attempted to get closer to that individual on foot for focal following. We confirmed a bird's identity by either reading its ID ring or, where this was impossible, establishing a line of sight coupled with a strong, directional radio signal. The positions of any individuals which could not be directly observed were determined by cross-triangulation where possible.

We spent a total of 48.25 hours radio-tracking over 10 days between 30 September and 15 October 2018. As birds were trapped and released throughout the study period, tracking effort varied substantially each day (median 2.67 hours per session, range 0.17–6.17 hours). Radio-tags were verified to be working on four of the six crows. One individual was never detected after release, possibly due to tag malfunction or the bird leaving the study area, and another tag was recovered detached in a garden seven days after the bird's release (the bird was subsequently seen alive and well). The remaining birds proved difficult to find and follow: only for 23 of a total of 199 radio fixes were we able to home-in on the location of the tagged bird and achieve a sighting. This was due to two main reasons: accessing many private properties in the study area proved difficult due to the presence of unrestrained guard dogs; and crows regularly crossed open sections of the river behind the residential area (20–100 m wide) and spent a large proportion of their time on small islands in the mangrove swamp of the river delta, which were inaccessible to us during the study period.

On one occasion, a crow was recorded feeding on *M. elengi* fruits, but no tool use or tool manufacture were observed throughout our radio-tracking study. After our tracking study had ended, we were able to briefly visit one of the small islands in the mangrove swamp, where we observed a radio-tagged crow (ID ring CE8) holding a tool as it flew through the canopy. We were unable to determine the tool type from this brief sighting.

Artifact-centered approach (see Fig. 1*D* in the main text)

Tool collection. Between 2012 and 2017, a total of 105 tools were collected in a private garden at site-3 by both researchers from our team and local residents (1). Tool collection was largely opportunistic, as NC crows visiting a bird water bath in one of the gardens would regularly leave tools behind after drinking. The "startle" method (9) was also used at least once, to distract a crow observed holding a tool. The time taken until tool recovery ranged from a few minutes (for the startle method) to possibly several days (for tools recovered opportunistically). In 2016 and 2017, tools were stored in re-sealable plastic freezer bags as soon as possible following their discovery, along with sufficient silica gel powder to cover them [n = 19; (10)]. Stored tools were transported to the UK for further analysis and archiving in the team's NC crow tool collection.

The recovered tools were all of broadly similar appearance [see Fig. 1*D* in the main text, and Figure 1C in (1)], exhibiting features characteristic of hooked stick tools collected elsewhere (a terminal hook, curved tool shaft, and bark stripped from the functional end). Most tools showed signs of foraging wear around the hook, but presented no morphological features that would have enabled reliable plant species identification [see main text, and (3, 9, 11)]. Having never observed hooked stick tool manufacture by wild crows at site-3, it was also not possible for us to recover and examine plant debris discarded by crows during raw-material processing. That said, some morphological features were preserved on a small subset of tools, including bark along the tool shaft with scars suggesting an alternate leaf arrangement and, less frequently, a small amount of leaf material or a small leaf bud on the non-functional end of the tool. While the appearance of tools suggested that they all originated from the same plant source, we were unable to identify the species through visual examination alone, despite also consulting several expert botanists [present study, and see also (1)].

DNA barcoding identifies candidate species. In May 2018, we selected for further analysis a subset of seven tools from the 19 tools collected at site-3 between 2016 and 2017 (see above). The first five tools were selected specifically to maximize our chances of successful DNA recovery, as they had fragments of bark remaining on the tool shaft. Once we had confirmed that DNA extraction was possible from these tools, we randomly selected an additional two tools with little bark remaining on the tool shaft, to diversify our sample. Given that visual inspection suggested all tools were of common material origin, and in order to preserve as much of our tool collection as possible for other research (since sampling was destructive), we limited our analysis to this subset of seven tools. Two subsamples were taken from each tool, and these were homogenized in 2 ml tubes with two tungsten beads using the FastPrep-24™ 5G Benchtop Homogenizer for up to one minute, or with QIAGEN Tissuelyser II for 6 minutes at 25 Hz. DNA extraction then proceeded according to the DNeasy Plant Mini Kit (QIAGEN, Manchester UK), except the lysis step was extended to 60 minutes.

For species identification, we amplified ~500 bp regions of the chloroplast *trnL-UAA* intron [*trnLc-d*; (12)] and a ~600 bp nuclear ribosomal region containing the internal transcribed spacer regions ITS1 and ITS2 [ITS5p-8p; (13)]. Amplification was in 20 µl reactions using approximately 100 ng of genomic DNA, 10 x reaction buffer (Bioline BIOTAQ Reagent Buffer), 30 nmol $MgCl_2$, 4 nmol dNTPs, 7.5 pmol primers, and Bioline BIOTAQ DNA polymerase (0.5 units). Negative controls and DNA extraction blanks were included in each PCR to check for potential contamination. Thermocycling was performed on a Bio-Rad Tetrad 2 (Bio-Rad, Hamburg, Germany) under the following conditions. For *trnL*: initial denaturation (94 °C for 4 min), followed by 35 cycles of denaturation (94 °C for 45 s), annealing (55 °C for 45 s) and extension (72 °C for 120 s), and a final extension of 10 min at 72 °C; and for ITS: initial denaturation (94 °C for 2 min), followed by 30 cycles of denaturation (94 °C for 60 s), annealing (55 °C for 60 s) and extension (72 °C for 90 s), and a

final extension of 5 min at 72 °C. Two independent PCRs were carried out for each subsample. Successfully amplified PCR products were cleaned using ExoSap-IT© (USB Corporation, Cleveland, Ohio, USA). Sequencing was resolved on an AB 3730xl Sequencer at Edinburgh Genomics (Scotland, UK). Sequences were checked and edited with reference to chromatograms using Sequencher v 5.4.1 (Gene Codes Corporation, Ann Arbor, MI, USA).

Sequence data were obtained for all seven crow tools (14 subsamples, with 2 replicates per tool), yielding identical haplotypes for all samples for both *trnL* and ITS. Sequences obtained for all tool samples were lodged with GenBank under accession numbers MT366813–MT366819 (*trnL*) and MT366951–MT366952, MT366955–MT366959 (ITS). There was no amplification in negative controls. The resulting sequence haplotype for each region was used for performing BLASTn searches against the National Center for Biotechnology Information Nucleotide nonredundant database (4 July 2018) to obtain a putative identification. Searches for both *trnL* and ITS indicated that the unknown plant material belonged to the family Sapotaceae, most likely the genus *Mimusops* or *Manilkara* (*trnL*: >98% identity; ITS: >95% identity). Specifically, the best 100 matches (excluding environmental samples) for *trnL* all possessed 98–99% identity and belonged to the Sapotaceae, with the most likely match being *Mimusops capuronii*, followed by *Manilkara zapota*. The ITS region showed similar results with all species in the top 100 matches belonging to the Sapotaceae and the unknown samples exhibiting 96–99% identity to *M. elengi*, followed by several species within this and one other genus (*M. caffra*, *M. zeyheri*, *M. comorensis*, *M. obovata*, *M. kummel*, and *Manilkara hexandra*) with 95–96% identity.

Based on these results, we carried out a qualitative comparison of the remaining tools with preserved samples of *M. elengi* (as the closest match and the only species of *Mimusops* native to New Caledonia) in three herbarium collections – the Royal Botanic Garden Edinburgh, Scotland [accessed physically and online; (14)], the Muséum National d'Histoire Naturelle, France [accessed online only; (15)], and the Institut de Recherche pour le Développement, New Caledonia [accessed online only through JSTOR Global Plants; (16)]. Morphological features preserved on a small subset of the tools (see above) were also observed in the terminal twigs of some *M. elengi* herbarium samples, but it was not possible at this stage to definitively confirm a match between these samples and the tools.

Material identity confirmed. In September 2018, we located and examined *M. elengi* trees at site-3 and in the surrounding area. Some terminal branches of these trees indeed appeared suitable for hooked stick tool making and possessed structural features we believe to be important in other plants used by NC crows [e.g., *D. virgatus*, Fabaceae; (2, 3, 17)]. These features include an acute branching angle less than 45°, a strong but flexible tool shaft, and a hook shaft of similar diameter to the tool shaft. In particular, we found "reiterations" – young epicormic shoots emerging from mature branches that replicate a tree's overall architecture (18) – on some *M. elengi* trees that appeared very similar in structure to the *D. virgatus* stems preferred by NC crows at site-2 (3).

Following these observations, we collected reference leaf samples of *M. elengi* (*n* = 3) and its closest locally occurring relative, *P. cinerea* (*n* = 2). Leaves were collected from separate trees at or near site-3 and site-1. In March 2019, DNA extraction, PCR amplification and sequencing for the reference samples were performed exactly as described above for the tool samples. The resulting DNA sequences for the reference data were lodged with GenBank under accession numbers MT366823–MT366824 (*trnL*) and MT366953–MT366954 (ITS) for *P. cinerea*, and MT366820–MT366822 (*trnL*) and MT366960–MT366962 (ITS) for *M. elengi*. Phylogenetic trees were generated using these samples plus the top 100 BLASTn matches (excluding environmental samples). Since taxa from both Sapotaceae and Theaceae were present in the top matches for *trnL*, *Acanthogilia gloriosa* (GenBank accession number EU348374), a polemonioid Ericale was included as the outgroup for *trnL*. Since all the taxa in the top matches for ITS were from the Sapotaceae, subfamily Sapotoideae, *Sarcosperma laurinum* (GenBank accession number AM408055), which has previously been shown to be sister to the rest of the family, was included as an outgroup for ITS (19).

Sequences were aligned using the MUSCLE algorithm (20). Maximum-likelihood (ML) trees were constructed using the .pml function in the package phangorn (21), implemented in R (22). The most appropriate model of DNA substitution was selected using the AIC and the function modelTest. We used the HKY model with the proportion of invariant sites and rate variation opti-

mized using the function optim.pml. Support for the branching topology was evaluated with 1,000 bootstrap replicates. Separate trees were generated for chloroplast (*trnL*) and nuclear (ITS) regions.

Sequence comparisons revealed that *M. elengi* reference material produced identical sequences to the tool samples for both DNA barcodes. The ML phylogenetic tree for *trnL* (data deposited in Dryad; see main text) showed poor resolution at the genus or species level within the Sapotaceae family (it showed an exact match between the *trnL* sequence of the *M. elengi* reference samples and the tool material, but this haplotype was also shared with a Madagascan species, *M. capuronii*, the only other *Mimusops* species with sequence data available). The ML tree for ITS produced the most informative data, with well-supported lineages largely corresponding to species/genera (data deposited in Dryad; see main text). The unknown plant material from tool samples clustered with sequences from species belonging to the *Mimusops* genus and was nested within a cluster of samples corresponding to *M. elengi*. The unknown tool samples had an identical ITS sequence to the New Caledonian *M. elengi* reference samples, and this sequence cluster was nested within a wider clade of *M. elengi* samples from Indonesia (GenBank accession number KF686246) and Thailand (GenBank accession numbers KF686245 and HF542849). Importantly, *M. elengi* is the only native *Mimusops* species in New Caledonia (23, 24), adding additional confidence to the identification. One other *Mimusops* species is recorded from New Caledonia as an introduction (*M. coriacea*); this shows a clearly distinct ITS sequence (GenBank accession number KM370965), which clusters separately to the tool samples and the reference material of *M. elengi*. The reference samples for *P. cinerea* clustered within the Sapotaceae, but outside the *Mimusops* genus as expected. A single *Manilkara hexandra* sequence from GenBank (GenBank accession number JX856473) is resolved within *Mimusops* in the ITS phylogeny (separate from the tool samples and *M. elengi*), although this is most likely a misidentification, as all other sequences from *Manilkara* clustered elsewhere in the tree. Therefore, based on an identical sequence match to reference material gathered from the study location, the unknown plant material used by NC crows to produce hooked stick tools at site-3 is identified as *M. elengi*.

Behavioral confirmation. In August and September 2019, 13 NC crows at site-1 were held on-site in temporary captivity for behavioral experiments, following the protocols described above. Birds were initially presented with a range of locally available plant material alongside extraction tasks in both their housing aviaries and in experimental chambers. The materials included two plant species known to have been used by crows at site-1 [*Acacia spirorbis* and *Melaleuca quinquenervia*; (7)], the two species flagged by our DNA barcoding analyses (*M. elengi* and *P. cinerea*), the preferred tool material of crows at site-2 [*D. virgatus*; (1)], as well as two other locally sourced plants with a structure judged suitable for hooked stick tool making.

Two of these crows made hooked stick tools (the others either made only non-hooked stick tools or did not make tools at all in captivity) and regularly chose *M. elengi* forks (in the presence of other plant species) to make these tools, using them to extract bait. This provided the first direct observations of NC crows processing *M. elengi* forks to manufacture hooked stick tools. Importantly, these tools manufactured (in captivity) from *M. elengi* by crows from site-1 appeared morphologically very similar to the tools we had previously recovered at site-3 (see above).

SI References

1. J. J. H. St Clair, B. C. Klump, J. E. M. van der Wal, S. Sugasawa, C. Rutz, Strong between-site variation in New Caledonian crows' use of hook-tool-making materials. *Biol. J. Linn. Soc.* **118**, 226–232 (2016).
2. J. J. H. St Clair, C. Rutz, New Caledonian crows attend to multiple functional properties of complex tools. *Phil. Trans. R. Soc. B* **368**, 20120415 (2013).
3. B. C. Klump, S. Sugasawa, J. J. H. St Clair, C. Rutz, Hook tool manufacture in New Caledonian crows: behavioural variation and the influence of raw materials. *BMC Biol.* **13**, 97 (2015).
4. B. Kenward, C. Rutz, A. A. S. Weir, J. Chappell, A. Kacelnik, Morphology and sexual dimorphism of the New Caledonian crow *Corvus moneduloides*, with notes on its behaviour and ecology. *Ibis* **146**, 652–660 (2004).
5. B. Heinrich, J. Marzluff, Age and mouth color in common ravens. *Condor* **94**, 549–550 (1992).

6. C. Rutz, J. Troscianko, Programmable, miniature video-loggers for deployment on wild birds and other wildlife. *Methods Ecol. Evol.* **4**, 114–122 (2013).
7. J. Troscianko, C. Rutz, Activity profiles and hook-tool use of New Caledonian crows recorded by bird-borne video cameras. *Biol. Lett.* **11**, 20150777 (2015).
8. L. A. Bluff, A. Kacelnik, C. Rutz, Vocal culture in New Caledonian crows *Corvus moneduloides*. *Biol. J. Linn. Soc.* **101**, 767–776 (2010).
9. G. R. Hunt, Manufacture and use of hook-tools by New Caledonian crows. *Nature* **379**, 249–251 (1996).
10. M. W. Chase, H. H. Hills, Silica gel: an ideal material for field preservation of leaf samples for DNA studies. *Taxon* **40**, 215–220 (1991).
11. G. R. Hunt, R. D. Gray, The crafting of hook tools by wild New Caledonian crows. *Proc. R. Soc. B* **271**, S88–S90 (2004).
12. P. Taberlet, L. Gielly, G. Pautou, J. Bouvet, Universal primers for amplification of three non-coding regions of chloroplast DNA. *Plant Mol. Biol.* **17**, 1105–1109 (1991).
13. M. Möller, Q. C. B. Cronk, Origin and relationships of Saintpaulia (Gesneriaceae) based on ribosomal DNA internal transcribed spacer (ITS) sequences. *Am. J. Bot.* **84**, 956–965 (1997).
14. Royal Botanic Garden Edinburgh, RBGE Herbarium catalogue (2020). Available at: https://data.rbge.org.uk/search/herbarium/
15. Muséum National d’Histoire Naturelle, MNHN Consultation des collections – Plantes vasculaires (2020). Available at: https://science.mnhn.fr/institution/mnhn/collection/p/item/search/form?lang=fr_FR
16. ITHAKA, Global Plants on JSTOR (2020). Available at: https://plants.jstor.org/
17. S. Sugasawa, B. C. Klump, J. J. H. St Clair, C. Rutz, Causes and consequences of tool shape variation in New Caledonian crows. *Curr. Biol.* **27**, 3885–3890 (2017).
18. P. B. Tomlinson, Tree architecture: new approaches help to define the elusive biological property of tree form. *Am. Sci.* **71**, 141–149 (1983).
19. K. E. Armstrong *et al.*, Patterns of diversification amongst tropical regions compared: a case study in Sapotaceae. *Front. Genet.* **5**, 362 (2014).
20. R. C. Edgar, MUSCLE: multiple sequence alignment with high accuracy and high throughput. *Nucleic Acids Res.* **32**, 1792–1797 (2004).
21. K. P. Schliep, phangorn: phylogenetic analysis in R. *Bioinformatics* **27**, 592–593 (2011).
22. R Core Team, *R: A Language and Environment for Statistical Computing* (R Foundation for Statistical Computing, 2011).
23. P. Morat *et al.*, Le référentiel taxonomique Florical et les caractéristiques de la flore vasculaire indigène de la Nouvelle-Calédonie. *Adansonia* **34**, 179–221 (2012).
24. J. Munzinger *et al.*, FLORICAL: Checklist of the vascular indigenous flora of New Caledonia (2020). Available at: http://publish.plantnet-project.org/project/florical
